# Temporin A and Bombinin H2 Antimicrobial Peptides Exhibit Selective Cytotoxicity to Lung Cancer Cells

**DOI:** 10.1155/2020/3526286

**Published:** 2020-06-26

**Authors:** Lucy Swithenbank, Phillipa Cox, Llinos G. Harris, Edward Dudley, Kathryn Sinclair, Paul Lewis, Floriana Cappiello, Claire Morgan

**Affiliations:** ^1^Swansea University Medical School, Institute of Life Science, Swansea University, Swansea SA2 8PP, UK; ^2^Department of Biochemical Sciences “A. Rossi Fanelli”, Sapienza University of Rome, Rome, Italy

## Abstract

**Background:**

Recently, antimicrobial peptides (AMPs) have been investigated for their use in cancer therapy. They have been reported to selectively target and kill cancer cells whilst leaving normal healthy cells unaffected. Certain Anura AMPs have expressed selective cytotoxicity against tumour cells.

**Aim:**

To test the potential of Anura AMPs bombinin H2, bombinin H4, temporin A, and temporin L for use as therapeutic agents for non-small cell lung carcinoma (NSCLC).

**Methods:**

Cytotoxic effects on NSCLC cell lines A549 and Calu-3 and normal epithelial cell line Beas-2B were tested using the CellTox Green Cytotoxicity Assay. Their haemolytic effects on human erythrocytes were also tested for their clinical relevance. Cell membrane profiling, using MALDI-TOF, was performed to ascertain if membrane characteristics of the NSCLC and Beas-2B cell lines may contribute to the AMPs mode of action.

**Results:**

Bombinin H4 (100–1.5 *μ*M, *p* < 0.05) and temporin A (100–50 *μ*M, *p* < 0.05) showed selective cytotoxicity towards the NSCLC cell lines. Furthermore, they exhibited low levels of haemolytic activity (bombinin H4, 0.061%; temporin A, 0.874%) comparable to untreated cells. Cell membrane profiling showed the phospholipid composition of normal epithelial cell line Beas-2B to be divergent from the cancerous cell lines. However, there was an overlap in the phospholipid profiles of the NSCLC cell lines supporting the hypothesis that the AMPs may have a selective affinity via the membrane composition of cancerous cell lines.

**Conclusion:**

These results suggest that bombinin H4 and temporin A show potential for application in lung cancer therapies. Further *in vitro* and *in vivo* studies are required to develop a greater understanding of their use as anticancer agents.

## 1. Introduction

In 2018, it was reported that there were an estimated 17 million new cancer cases and 9.6 million cancer related deaths worldwide, with lung cancer being the most common cause of death [1]. Lung cancer is commonly treated via chemotherapy [2]. However, the success of this treatment is limited due to the rise of chemotherapeutic resistance in cancer cells due to alterations occurring within membrane transporter proteins such as multidrug resistance (MDR) proteins and P-glycoprotein. Furthermore, the nonspecific targeting of the chemotherapy agents results in their toxicity towards healthy dividing cells, causing deleterious side effects such as nausea, diarrhoea, hair loss, anaemia, and infections, all of which reduce the patients' overall quality of life. Unfortunately, the prognosis of lung cancer patients has not shown the same level of improvement over recent years as has been observed in other cancers such as breast and prostate [3]. Following an average of 4–6 months' posttreatment initiation, patients suffer disease progression, highlighting the long term ineffectiveness of the current treatments and aggressiveness of the disease [4]. The 5-year survival rate for non-small cell lung cancer (NSCLC) is currently only 10–20% [5]. It is therefore imperative that new drug treatments that have neither the toxicity nor the mechanisms of resistance associated with conventional chemotherapy are developed.

Antimicrobial peptides (AMPs) are a group of compounds that are a conserved element of the innate immune response [6], found in all species investigated such as bacteria, fungi, plants, insects, birds, fish, amphibians, and mammals [6, 7]; these small peptides are between 12 and 50 amino acids long [8]. They have been shown to effectively kill a wide range of micro-organisms such as viruses, fungi, and both Gram-positive and -negative bacteria [9]. AMPs are grouped into two sets: those which are toxic to bacteria and cancer cells but not to noncancerous mammalian cells and those which are toxic towards all three cell types [10]. AMPs specific selectivity for cancer cells over normal epithelial cells is generally attributed to several complimenting characteristics between the cancer cell membranes and the AMPs. Such characteristics include their ability to interact electrostatically with each other, hydrophobicity of the AMPs represented as therapeutic index, and the sequence of the AMP. The electrostatic interaction between the AMP and the target cell membrane is one of the key drivers of interaction, with AMPs typically being cationic with a net positive charge between +2 and +7 due to an excess of basic amino acids such as arginine, lysine, and histidine [11]. Not all AMPs possess anticancer properties but those that do, anticancer peptides (ACPs), have been recognised as being more specific in their effects, producing less harmful side effects than chemotherapy and traditional therapies [10, 12, 13].

AMPs found in Anura frog skin secretions have been used medicinally for many years. The dorsal skin secretions collected from Anura are amongst the richest sources of naturally formed AMPs, acting as part of the defence mechanisms to protect the frog skin from pathogen invasion and predator ingestion [14]. The Anura expels the AMPs, along with other molecules such as alkaloids, neuropeptides, and biogenic amines, in large amounts in response to threat or injury [15]. Each Anura species secretes a unique set of AMPs which leads to the variety in properties such as antimicrobial, anticancer, and haemolytic effects [16]. Certain Anura AMPs have been observed to express selective cytotoxicity against tumour cells, such as AMPs from African claw frogs from the Pipidae family which have demonstrated tumouricidal properties against small cell lung cancer cell lines [17] and bladder cancer cell lines [18] as well as a range of hematopoietic cell lines [19].

The purpose of this study is to investigate antimicrobial peptides from the Anura species *Rana temporaria* (temporin A and temporin L) [20] and *Bombina variegata* (bombinin H2 and bombinin H4) [21] to determine whether they display selective cytotoxicity towards lung cancer cells, thus offering potential as new chemotherapeutic agents to treat lung cancer. The percentage of cell viability with AMPs at varying concentrations and time points was tested using lung cancer cell lines (A549 and Calu-3) as well as noncancer cell line (Beas-2B). Furthermore, cell membrane characterisation was analysed to determine if membrane composition may affect the AMPs mode of action.

## 2. Materials and Methods

### 2.1. Peptides

Temporin A [FLPLIGRVLSGIL], temporin L [FVQWFSKFLGRIL], bombinin H2 [IIGPVLGLVGSALGGLLKKI], and bombinin H4 [I–(D-allo-1) GPVLGLVGSALGGLLKKI] were synthesized by Selleck Chemicals (Houston, TX, USA).

### 2.2. Peptide Validation

Matrix assisted laser desorption/ionisation (MALDI) mass spectrometry analysis was carried out on the samples following the addition of 100 *μ*l of 0.1% formic acid to solubilise the peptide. The matrix solution was made up of *α*-cyano-4-hydroxycinnamic acid (10 mg/ml) combined with a 50 : 50 solution acetonitrile and 0.1% trifluoroacetic acid. When all the samples were dry, the MALDI plate was loaded into the Voyager-DE STR Biospectrometry Workstation (Applied Biosystems, Framingham, MA). Data acquisition parameters were set: ion mode, positive; instrument mode, reflector; instrument range, 1000 to 4000 Da; low mass gate, 1000 Da; total scans, 100 shots per spectrum; accelerating voltage, 20,000 V. The results were analysed using Voltage Data Explorer^TM^ version 4.0 (Applied Biosystems, Framingham, MA). The spectrum was calibrated against the reference proteins (angiotensin, bradykinin, and neurotensin).

Electrospray ionisation (ESI) mass spectroscopy was used in combination with MALDI mass spectroscopy to further validate the peptide identities. Each peptide was mixed with 100 *μ*l of methanol before being pipetted into the LTQ Orbitrap XL (Thermo Fisher Scientific, Waltham, MA). The peptides were fragmented in higher-energy collisional dissociation (HCD) mode with an isotope band width of 1.5 m/z.

### 2.3. Antimicrobial Properties

*Staphylococcus epidermidis* 1457 and *S. epidermidis* 5179-R1 were cultured on horse blood agar plates for 24 hours at 37°C and subsequently used to inoculate 3 ml of prewarmed Tryptic Soy Broth (TSB; Becton Dickinson, Cockeysville, USA). Precultures were grown in a shaking incubator for 2–3 hours at 37°C and used to inoculate 200 *μ*l fresh TSB in 96-well tissue culture plates (NUNC, Thermo Fisher Scientific, Roskilde, Denmark) to a starting OD_600_ of 0.05. To each test well, 100 *μ*M of peptides was added. This concentration was chosen as it was the upper limit of the concentrations being investigated for anticancer potential. Untreated bacteria were used as controls.

The 96-well plate was inserted into a BMG FLUOstar Omega microplate reader (BMG LABTECH, Germany) set to a wavelength of 600 nm. The OD of each well was read every hour over 24-hour period, to determine the effect of the AMPs on the growth rate/population density of the bacteria. All experiments were performed in triplicate, and each strain and condition tested in triplicate.

### 2.4. Cell Culture

NSCLC cell line A549 (ATCC–CCL185) and normal epithelial cell line Beas-2B (ATCC CRL-9609) were routinely grown in DMEM (Corning, Thermo Fisher Scientific, Basingstoke, UK) supplemented with 10% foetal bovine serum (FBS) (South American Origin, Biosera, Uckfield, UK), 2 mM L-glutamine (Gibco, Thermo Fisher Scientific, Basingstoke, UK), and 1% penicillin/streptomycin (Gibco, Thermo Fisher scientific, Basingstoke, UK). The NSCLC cell line Calu-3 (ATCC HTB55) was routinely grown in RPMI-1640 (Corning, Thermo Fisher Scientific, Basingstoke, UK) supplemented with 10% FBS, 2 mM L-glutamine, and 1% penicillin/streptomycin. All cells were incubated at 37^o^C and 5% CO_2._

### 2.5. Cytotoxicity Assays

#### 2.5.1. CellTox Green Cytotoxicity Assay

The CellTox Green Cytotoxicity Assay was used to determine reduction in membrane integrity due to AMP treatment. CellTox Green Dye was mixed with cell stock made in phenol-free, serum-free medium to a ratio of 1 *μ*l dye: 1000 *μ*l cell stock. Phenol-free medium decreases the background noise of the fluorescence reading so that a more accurate reading can be taken. This solution was then used to dilute the peptide to specific concentrations (100 *μ*M, 50 *μ*M, 25 *μ*M, 12.5 *μ*M, 6.25 *μ*M, 3 *μ*M, 1.5 *μ*M). Once the cells were dosed, the plates were maintained at a temperature of 37°C and 5% CO_2_. Untreated cells, comprising media and CellTox Green Dye, were used as a control; cell lysis buffer (provided in the CellTox Cytotoxicity Assay kit) was used as a positive control, indicating cell death.

Fluorescence readings were taken using a POLARstar Omega plate reader (BMG LABTECH, Germany) at 24 hours in accordance with published literature [22, 23]. The plate was read from the bottom optic at 485–510 nm excitation and 520–530 nm emission, with gain adjusted to 1500 OD. The gain was optimised so that the maximum value was not reached by any of the peptides for any cell lines over the 24 h treatment. Readings were corrected against the blank average. Only the peptides which were cytotoxic to the NSCLC cell lines, A549 and Calu-3, were tested for selectivity on the normal lung epithelial cell line, Beas-2B. All experiments were performed in triplicate.

#### 2.5.2. Haemolytic Assay

The HaemoScan Biomaterial Haemolytic Assay (HaemoScan, Netherlands) was used to investigate the cytotoxicity of the AMPs on human erythrocytes. Only the AMPs which displayed selective cytotoxicity towards the cell lines A549 and Calu-3 were tested for haemolytic properties.

The protocol of the assay was followed according to the manufacturers' instructions. In summary, the erythrocyte suspension was prepared by adding 5 ml of the supplied wash buffer to the erythrocyte suspension and centrifuging at 400 × g for 10 minutes. The supernatant was then removed. This step was repeated twice with the wash buffer, and the steps were then repeated replacing wash buffer with dilution buffer twice more. The final pellet was then suspended in 5 ml of dilution buffer to form a colourless supernatant.

The minimum concentration of each peptide that had a significant effect on A549 and/or Calu-3 without significantly effecting noncancer cells (Beas-2B) was added to 0.5 ml of erythrocyte suspension (bombinin H4, 6.25 *μ*M; temporin A, 50 *μ*M). A haemoglobin calibration curve was also produced to establish the haemoglobin concentrations present in the treatment groups of the erythrocyte suspension after exposure to the AMPs.

Both the calibrator and treatment groups were then incubated at 37°C for 24 hours. After 24 hours of incubation the samples were centrifuged at 4000 × g for 1 minute and 20 *μ*l of the supernatant pipetted into a 96-well plate along with 180 *μ*l of assay buffer. The plate was agitated on a plate shaker before being read on a BMG FLUOstar Omega microplate reader (BMG LABTECH, Germany). The absorbance was read at three wavelengths, 380, 415, and 450 nm (Harboe method), and the calculation (2 × 415)–(450 + 380) was applied to give a final OD.

Haemoglobin concentrations of the test samples were then established by comparing the treatment readings against the haemoglobin calibration curve. The haemoglobin concentration of the treatment groups was presented as a percentage of the total haemoglobin present in the sample, expressed as percentage haemolysis. All experiments were performed in triplicate.

### 2.6. Cell Membrane Characterisation

#### 2.6.1. Phospholipid Extraction and MALDI-TOF MS Analysis

Prior to phospholipid analysis, cultured cell lines were harvested as follows: culture medium was discarded from the flasks; the cells were washed with 10 ml PBS and were subsequently incubated with 3 ml 0.025% trypsin/EDTA for 3–5 min. The reaction was terminated by the addition of 10 ml cell culture medium, and the samples were centrifuged at 10,000 × g for 5 min.

Cell pellets were then solubilised in 200 ul of chloroform: methanol (2 : 1) and then diluted (1 : 5) in either dihydroxybenzoic acid (DHB) at a concentration of 10 mg/ml in methanol containing 0.1% trifluoroacetic acid or para-nitroaniline (PNA) at a concentration of 10 mg/ml in chloroform: methanol (2 : 1). One microliter of each mixture was then spotted on a clean MALDI plate (Applied Biosystems). Samples were analysed using a Voyager-DE STR, MALDI-TOF mass spectrometer (Applied Biosystems, Framingham, MA). A 337 nm specific laser wavelength was used to ensure that only the outer membrane was detected. Data acquisition parameters were set: time delay of 200 nanoseconds, total scans of 100 shots per spectrum, and accelerating voltage of 20,000 V. The two matrices (DHB and PNA) were utilised and run in both the positive and negative modes, respectively. The m/z signal was aligned across each sample within 0.5 of each other using Excel. The results were analysed and normalised using Voltage Data Explorer^TM^ version 4.0 (Applied Biosystems, Framingham, MA). Any background noise was removed, m/z values between 1 and 1600 were collected, and new spectra were established.

#### 2.6.2. Identification and Charge of Phospholipids

Possible identities of each membrane component at specific m/z values were assigned according to the work of Estrada and Yappert [24], e.g., PC (40 : 6), which represents the head-group (number of carbons in the fatty acid chain: number of double bonds).

### 2.7. Statistical Analysis

Intragroup data readings were checked for outliers using the Grubbs test, identifying outliers with a *p* value greater than 0.05 when compared with the expected distribution of results. Any outliers identified were excluded from further statistical analysis. A one-sample Kolmogorov–Smirnov (K–S) test was carried out on each data group to test the normality of the data to determine appropriate further data analysis tests. All data was shown to be of a non-normal distribution and therefore a Kruskal–Wallis nonparametric analysis was carried out. To identify any significant differences between groups, a Mann–Whitney test was used to test individual treatment groups against the untreated control group. A *p* value of <0.05 was considered significant.

## 3. Results

MALDI and ESI mass spectrometry confirmed that the synthetically made identities of each peptide accurately represented the peptide found from the natural source, Anura bombinin H2 and its diastereomer bombinin H4, containing a D-alloisoleucine at the second N-terminal residue as a result of a posttranslational modification, temporin A, and temporin L [25].

### 3.1. Antimicrobial Properties

**Figure 1:**
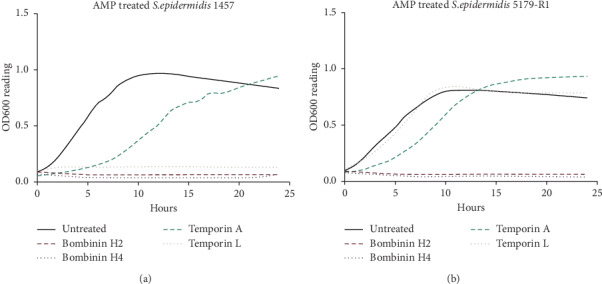
(a) Effects of the AMPs on *Staphylococcus epidermidis* 1457 over a 24-hour treatment period. (b) Effects of the AMPs on *Staphylococcus epidermidis* 5179-R1.

Untreated *S. epidermidis* 1457 and 5179-R1 underwent rapid proliferation throughout the initial 8 hours, reaching a maximum OD_600_ of 0.95 and 0.7, respectively, after 10 hours before entering a stationary phase. For *S. epidermidis* 1457, bombinin H2, bombinin H4, and temporin L (100 *μ*M) significantly inhibited proliferation over the 24 hours (*p* ≤ 0.001; Figure 1), whilst only bombinin H2 and bombinin H4 (100 *μ*M) significantly inhibited growth of *S. epidermidis* 5179-R1 (*p* ≤ 0.001; Figure 1(b)).

### 3.2. Cell Cytotoxicity

**Figure 2:**
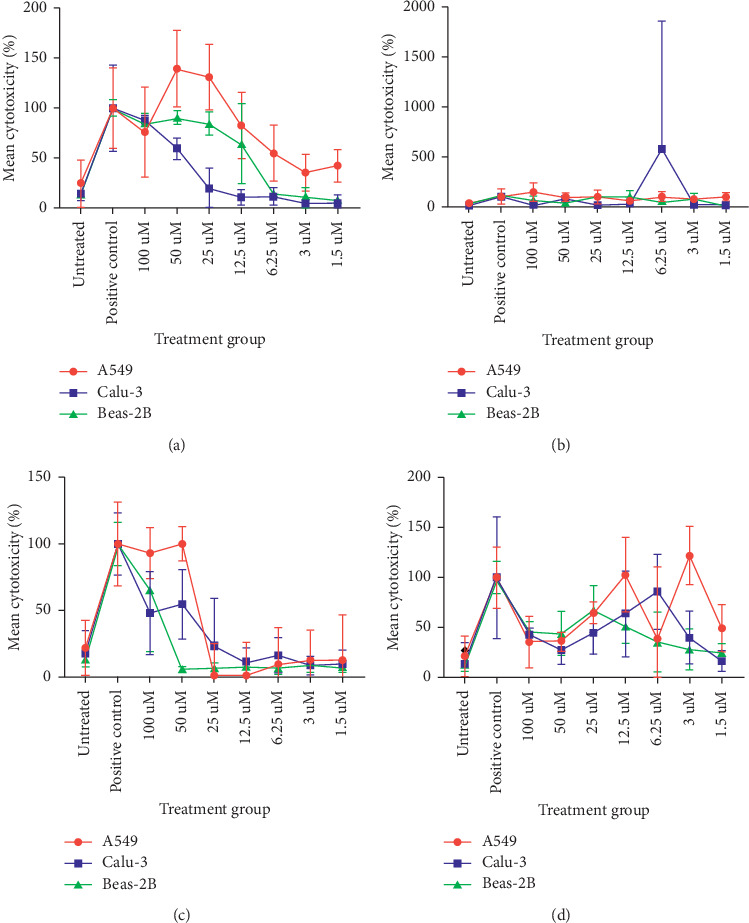
The cytotoxic effects observed on A549, Calu-3, and Beas-2B cell lines induced by (a) bombinin H2, IC50 value: 5.632 × 10^−7^ (95% CI 3.935 × 10^−7^ to 7.9 × 10^−7^); (b) bombinin H4, IC50 value: 5.637 × 10^−7^ (95% CI 3.935 × 10^−7^ to 7.9 × 10^−7^); (c) temporin A, IC50 value: 5.637 × 10^−7^ (95% CI 3.935 × 10^−7^ to 7.9 × 10^−7^); and (d) temporin L, IC50 value: 5.632 × 10^−7^ (95% CI 3.935 × 10^−7^ to 7.9 × 10^−7^).

When bombinin H2 was applied to the A549 cell line, statistical analysis showed significant cell death between the untreated cells and the treated cells at concentrations ranging from 12.5 *μ*M to 50 *μ*M (*p* ≤ 0.05). For Calu-3 cells, bombinin H2 resulted in significant cell death, when compared to the control, at concentrations of 50 *μ*M and 100 *μ*M (*p* ≤ 0.001) (Figure 2(a)). However, significant cell death also occurred when the noncancerous cell line Beas-2B was treated with bombinin H2 at concentrations of 12.5 *μ*M to 100 *μ*M. These data show that bombinin H2 is not selectively cytotoxic to cancer cells.

Bombinin H4 proved to be highly toxic to the A549 cell line after 24-hour exposure, with significant cell death being observed from 1.5 *μ*M to 100 *μ*M (*p* ≤ 0.05). However, in the Calu-3 cell line, significant cell death was only observed in the higher concentrations, 50 *μ*M and 100 *μ*M (*p* ≤ 0.001), when compared to the untreated cells (Figure 2(b)). In the Beas-2B cell line, significant cell death was observed at 12.5 *μ*M–100 *μ*M (*p* ≤ 0.05). As bombinin H4 showed selective cytotoxicity to A549 cells at the lowest concentration tested but not in the Beas-2B cells, it is suggested that bombinin H4 exhibits some level of selective cytotoxicity.

Interestingly, temporin A showed a dual effect on the A549 cell line. At concentrations of 50 *μ*M and 100 *μ*M (*p* ≤ 0.001), significant cell death was observed. However, at concentrations of 3 *μ*M–25 *μ*M, a significant increase in cell viability was observed (*p* ≤ 0.050). For Calu-3 cells, 50 *μ*M and 100 *μ*M temporin A induced significant cell death (*p* ≤ 0.01) when compared to the untreated cells but, unlike A549, did not increase cell viability at the lower concentrations (Figure 2(c)). When temporin A was applied to the Beas-2B cell lines, significant cell death was only observed at 100 *μ*M (*p* ≤ 0.05). From these results, it could be suggested that temporin A is selectively cytotoxic at 50 *μ*M.

The A549 cell line treated with temporin L at concentrations of 1.5 *μ*M–25 *μ*M (*p* ≤ 0.05) resulted in significant cell death when compared to the untreated cells. In the Calu-3 cell line, temporin L induced significant cell death across the whole range of concentrations, 1.5* μ*M–100 *μ*M (*p* ≤ 0.05), when compared to the untreated cells (Figure 2(d)). As with Calu-3 cell line, Beas-2B cell line showed significant cell death across the whole temporin L concentration range, 1.5 *μ*M–100 *μ*M (*p* ≤ 0.05), when compared to the untreated cells. Temporin L, therefore, was shown not to be selectively cytotoxic.

### 3.3. Haemolytic Properties

In the previous cytotoxicity investigations, only bombinin H4 and temporin A were identified to have selective cytotoxicity potential towards lung cancer cells, A549, and/or Calu-3 cell types, over noncancerous cell line Beas-2B. Therefore, due to their selective cytotoxic characteristics, only bombinin H4 and temporin A were investigated for haemolytic activity after 24 h treatment at 6.25 *μ*M and 50 *μ*M, respectively. Interestingly, whilst bombinin H4 did not produce a cytotoxic effect on erythrocytes, levels of haemolysis were significantly less than untreated cells (*p* ≤ 0.001), suggesting that bombinin H4 may have a protective effect on erythrocytes viability. Temporin A, though nontoxic to erythrocytes with a haemolysis value comparable to untreated cells (*p* ≤ 1.000), did not display the same protective attributes as bombinin H4 (Table 1).

### 3.4. Cell Membrane Characterisation

**Figure 3:**
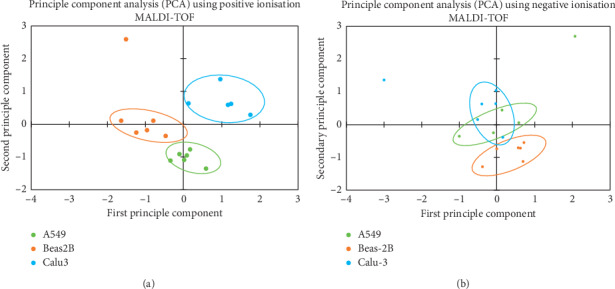
(a) PCA scatterplot of normalised MALDI-TOF DHB derived data. Phospholipid spread for six repeats of three *in vitro* cell lines is shown (A549, Beas-2B, Calu-3). The phospholipid profiles of each cell line appear to be clearly distinct from one another. (b) The relationship between six repeats for three *in vitro* cell lines via PCA is shown. There is an overlap in the phospholipid expression between he cancerous cell lines, whereas the normal cell line was separate from the cancerous cells.

The phospholipid profile of each cell line membrane was studied in order to understand the possible differences it presents to an AMP and hence the potential impact of membrane structure on AMP cytotoxic activity. Principle component analysis (PCA) using positive ionisation MALDI-TOF (Figure 3(a)) showed that Beas-2B, A549, and Calu-3 cell lines were distinct from one another whilst staying clustered within their cell type, indicating that membrane phospholipid expression was different among all three cell lines. Negative-mode MALDI-TOF principle component analysis showed the phospholipid composition of normal epithelial cell line Beas-2B to be divergent from the cancerous cell lines. However, we found that there was an overlap of the phospholipid profiles of the A549 and Calu-3 cancer cell lines (Figure 3(b)) which may help explain why the AMPs had a similar effect upon the cancerous cell lines but reacted differently with the noncancerous Beas-2B.

Membrane phospholipid expression profiles were also compared to try and identify any differences in cell membrane composition, which may account for the different effect the AMPs had on cell toxicity. When comparing the normal epithelial cell line Beas-2B with the NSCLC cell line A549, twenty-four significant peaks were acquired where the phospholipid was more abundant in either Beas-2B or A549 (*p* < 0.05); thirteen of the twenty-four had a *p* value of <0.005 (Figure 3(b)). When comparing Beas-2B cell line with NSCLC cell line Calu-3, thirty-two membrane components were found to be significantly different (*p* < 0.05) with twenty of those having a *p* value of 0.005.

**Figure 4:**
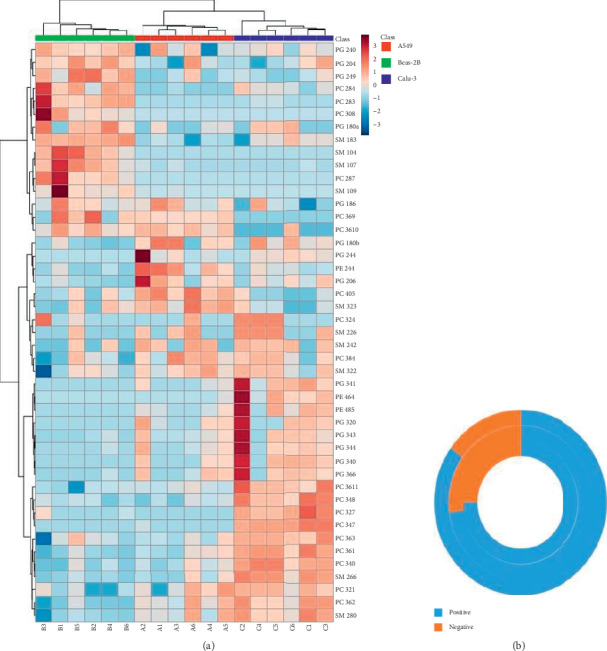
(a) The relative intensity of the phospholipids showing differences between the cell lines, obtained via positive-mode MALDI-TOF. (b) The relative percentage of positive and negative phospholipids deemed as significantly different between the outer leaflets of the three cell lines.

The variation in abundance of the phospholipids between cell lines (Figure 4) represents how the presence affects the overall cell membrane charge. The average relative intensities (%) of the identified lipid species were allocated their corresponding charge, the abundance of which was used to create a percentage of negative and positive phospholipids within each cell line. Beas-2B cells had a greater percentage of positive phospholipids and fewer negative phospholipids in comparison to A549 and Calu-3 cell lines (*p* < 0.05). When Calu-3 and A549 cells were compared, Calu-3, the stage III cancer cell line, was found to be more negatively charged (1.7%) than the A549 cell line.

## 4. Discussion

Cancer treatments can vary: surgery, chemotherapy, radiotherapy, or a combination of these treatments. With specific regard to lung cancer, 90% of individuals with small cell lung cancers will initially respond to chemotherapeutic treatment, but almost all will relapse with multidrug resistant disease, whilst patients with non-small cell lung cancer have a low response rate to chemotherapy due to inherent drug resistance [26]. In recent years, AMPs have attracted attention as potential anticancer drugs due to reports that they can selectively target and kill cancer cells whilst leaving normal healthy cells unaffected, thus making them attractive candidates for investigation. AMPs found in Anura have been used for medicinal purposes for years, yet only a handful of studies have reported the cytotoxic effects of Anura AMPs against different cancer types [17, 18]; therefore, we sought to determine if the AMPs bombinin H2, H4 (from the Anura species *Bombina variegate*) and temporin A and L (from the Anura species *Rana temporaria*) displayed selective anticancer activity, thus offering potential as new chemotherapeutic agents to treat lung cancer.

In this study we have been able to demonstrate that temporin A and bombinin H4 interacted more effectively towards the NSCLC cell lines than in the normal lung cell line. Temporin A showed the greatest potential as an anticancer agent due to its ability to initiate significant cell death in both NSCLC cell lines, at the same concentration, without significant cell death in the Beas-2B cell line. Interestingly, bombinin H4 selectively killed A549 cells but not Calu-3 cells. The difference in the ability of bombinin H4 to induce cytotoxic activity in the cancer cell lines suggests a difference in the cell membrane protein composition of the two cancer cell lines as a possible explanation for this observed effect. The membrane phospholipid profile demonstrated that the cell lines clustered into distinct groups, suggesting that the cells could have a distinct phospholipid compositional signature on their outer leaflets. However, upon investigation of the outer membranes in negative-mode MALDI-TOF, PCA showed that whilst Beas-2B cells have a distinct phospholipid signature from that of the cancerous cells, the cancerous cells overlapped, implying that the cancer cell lines have similar phospholipid profiles. Thus, cell membrane phospholipid composition, alone, cannot explain why bombinin H4 was cytotoxic to A549 cells but not Calu-3.

The charge of the cell membrane can also affect the affinity of the AMP for selective targeting of cancerous cells. As expected, we found that normal epithelial Beas-2B cells had a greater percentage of positive phospholipids and fewer negative phospholipids in comparison to the A549 and Calu-3 cell lines. Although PCA could not identify specific differences between the cell lines, normal mammalian cells predominantly contain zwitterionic phospholipids within their outer leaflet such as PC and SM, whilst negatively charged phospholipids such as PS and PG reside on the inner leaflet. In contrast, in cancer the negatively charged phospholipids become externalised to the outer leaflet [27–30], enhancing electrostatic interaction between the AMPs and cancer cells.

When A549 and Calu-3 phospholipid chargers were compared, we found Calu-3, the stage III cancer cell line, to be more negatively charged (1.7%) than the A549 cell line. Given that AMPs exert their cytotoxic effects through electrostatic interaction, it is surprising that the more negatively charged Calu-3 cells were not as susceptible to bombinin H4 as the A549 cells.

Thus, another factor that needs to be taken into consideration is the properties of the AMPs themselves. Temporin A was cytotoxic to both cancer cell lines at the same concentration, yet bombinin H4 induced cell death at much lower concentrations in A549 than Calu-3. Thus, it may be argued that the peptide sequence of the AMP, itself, plays a major role in its cytotoxic potential. Indeed, the sequence of an AMP can affect its ability to interact with cell membranes. Several early studies have demonstrated that peptide helicity is important for toxicity [31, 32]. Incorporation of D-amino acids into cell lytic peptides causes a reduction in their cytotoxicity on mammalian cells [33, 34]. Bombinin H4 has single L-D-isomerization, which may be enough to reduce the peptides alpha-helical content, in turn reducing its ability to bind to cell membranes. The disruption of the alpha-helical content can lead to the disruption of the hydrophobic face of the AMP with the positively charged residue on the cell membrane [35]. This in turn could prevent the binding of the AMP to the membrane from weakened polar attraction. This structural change, in combination with a slightly lower positive charge on the Calu-3 membrane, may help explain why bombinin H4 was not as cytotoxic to this cell line.

Many studies investigating the anticancer properties of AMPs use cell lines from different cancer types [12, 36, 37]; our study highlights the importance of investigating several cancer cell lines, especially from the same cancer type, to determine if any observed AMP anticancer effect is consistently observed across multiple cell lines or is cell line specific.

For AMPs to be viable anticancer agents they must not induce haemolytic responses in the patient. Temporin A and bombinin H4 were tested on human erythrocytes to investigate their haemolytic properties at the minimum concentrations required to cause significant cell death in NSCLC cell lines, A549 and Calu-3. Encouragingly, for temporin A, we observed a haemolysis value comparable to untreated cells. With bombinin H, haemolysis was significantly less than the untreated control cells, suggesting that it actually had a protective effective on erythrocyte viability. The membrane of erythrocytes is asymmetrically distributed with glycolipids, phosphatidylcholine, and sphingomyelin expressed on the outer membrane to form a hydrophilic environment. The surface charge of erythrocytes has been shown to have a zeta potential of 10 mV, which is considered to be a neutral charge [38]. Thus, the neutral charge of the erythrocytes may not attract the predominantly positively charged AMPs. Furthermore, the relative size of the cell lines compared to the erythrocytes should also be considered. The cell lines assessed have a diameter of 90–240 *μ*M, whereas the erythrocytes have a diameter of 6-8 *μ*M [39, 40]. Therefore, it could be suggested that the erythrocytes are too small to attract the AMP, preventing haemolysis from occurring.

It is generally accepted that the selective cytotoxicity of AMPs towards bacteria and cancer cells is due to both cell types possessing a negative charge on their cell membranes. Therefore, we sought to investigate if antimicrobial activity could be used as an indicator of an AMPs anticancer potential. Bombinins H2 and H4 showed significant antimicrobial activity towards *S. epidermidis* 1457 and 5179-R1 at 100 *μ*M, whilst temporins A and L exhibited some antimicrobial effects on the bacteria but not to the same extent as the bombinins. Given that bombinin H2 and temporin L were not selectively cytotoxic and temporin A demonstrated the most efficacious anticancer activity, the antimicrobial effect of an AMP cannot be used as an indicator for its anticancer potential.

## 5. Conclusion

Research on the anticancer properties of AMPs has increased in recent years. Many of these studies use different cancer cell lines but usually only one from a particular cancer type. Here we show that the effectiveness of AMPs as anticancer agents varies not just between AMPs but also between cancer types, even within a particular cancer type. Therefore, it is imperative that studies are conducted using more than one cell line from the same cancer type to ascertain if an AMP cytotoxicity is cell line specific. Furthermore, our preliminary investigation of four Anura AMPs demonstrated that temporin A and bombinin H4 may hold potential as anticancer agents, and further *in vitro* and *in vivo* work is now required to fully ascertain their anticancer potential and mode of action.

## Figures and Tables

**Table 1:** The mean percentage haemolysis induced by AMPS bombinin H4 and temporin A.

Treatment group	Mean percentage haemolysis (%)
Untreated cells	0.8252
Total haemolysis	100
Bombinin H4	0.0606
Temporin A	0.8739

## Data Availability

All data generated or analysed during the current study are included in this published article.
